# A method of percutaneous vertebroplasty under the guidance of two C-arm fluoroscopes

**DOI:** 10.12669/pjms.302.4099

**Published:** 2014

**Authors:** Ren-Jie Xu, Yong-Qing Yan, Guang-Xiang Chen, Tian-Ming Zou, Xiao-Qiang Cai, Dong-Lai Wang

**Affiliations:** 1 Ren-Jie Xu, MD, Department of Orthopaedics, Suzhou Municipal Hospital, Suzhou 215002, China.; 2 Yong-Qing Yan, MD, Department of Orthopaedics, Ningbo No.2 Hospital, Ningbo 315010, China.; 3 Guang-Xiang Chen, M.Sc., Department of Orthopaedics, Suzhou Municipal Hospital, Suzhou 215002, China.; 4 Tian-Ming Zou, M.Sc., Department of Orthopaedics, Suzhou Municipal Hospital, Suzhou 215002, China.`; 5 Xiao-Qiang Cai, M.Sc., Department of Orthopaedics, Suzhou Municipal Hospital, Suzhou 215002, China.; 6 Dong-Lai Wang, MD, Department of Orthopaedics, Suzhou Municipal Hospital, Suzhou 215002, China.

**Keywords:** Osteoporotic vertebral compression fractures, Percutaneous vertebroplasty, Two C-arm fluoroscopes

## Abstract

***Objective:*** To compare the clinical application in the percutaneous vertebroplasty under the guidance of one or two C-arm fluoroscopes.

***Methods:*** One hundred forty three elderly patients with Osteoporotic vertebral compression fractures (OVCFs) underwent percutaneous vertebroplasty under the guidance of one or two C-arm fluoroscopes. The number of pulsed imagings, the time of operation and the incidence of cement leakage were recorded.

***Results:*** The average number of pulsed imagings was 16.00±1.58 vs 13.07±2.00 per patient under the guidance of one vs two C-arm fluoroscopes. The average time of operation was 48.42±5.00 minutes vs 39.70±7.42 minutes per patient under the guidance of one vs two C-arm fluoroscopes. The incidence of cement leakage was 20% vs 15.7% of the patients under the guidance of one vs two C-arm fluoroscopes. The differences in the number of pulsed imagings and the time of operation were statistically significant. The difference in incidence of cement leakage was not statistically significant.

***Conclusion:*** The two-fluoroscopic technique reduce the labor cost, the radiation, the time of operation and the operation risk.

## INTRODUCTION

Osteoporosis is the most common metabolic disorder of bone among the ageing population worldwide.^1^ Several fracture types are common with osteoporosis, including vertebral compression and fractures of the hip, femoral neck, intertrochanteric femur, distal radius, proximal humerus, distal femur, and proximal tibia.^2^ Osteoporotic vertebral compression fractures (OVCFs) are the most common fragility fractures, exceeding even hip and wrist fractures in incidence. An estimated amount of 1.4 million OVCFs received clinical attention worldwide in the year 2000.^3^ Percutaneous vertebroplasty (PVP) is a therapeutic, interventional radiologic procedure that involves injection of acrylic cement (most commonly polymethylmethacrylate/PMMA) into a vertebral body lesion for the strengthening of bone and the relief of pain. Fluoroscopy is used to guide needle and sheath placement, and a mixture of polymethylmethacrylate (PMMA) and barium is injected under direct fluoroscopic visualization. During the procedure guided by a single C-arm X-ray machine, the C-arm need to be rotated for either antero-posterior (AP) or lateral radiographic view, that might increase the radiation wasted during readjusting to the second plane of view and be time-consuming. Biplane fluoroscopy is preferred because it allows orthogonal, real-time visualization for cannula introduction and cement injection. However, this equipment is expensive and not commonly available in hospitals in China. An alternative is to use two C-arm fluoroscopes. 

The aim of this study was to compare the efficacy of performing PVP using one or two C-arm fluoroscopes.

## METHODS

This was a prospective cohort study of patients with OVCFs who were treated at the Department of Orthopaedics, Suzhou Municipal Hospital. Between September 2009 and April 2012, one hundred forty three consecutive patients (102 female and 41 male, mean ages: 74.0 years, range 52, 91) with single vertebrae who underwent PVP under the guidance of two C-arm fluoroscopes by one surgeon were included in the study after Institutional Review Board approval. The hospital’s ethical committee approved the protocol. Participants received the IRB-approved protocol and informed consent, detailing all aspects of the study and the withdrawal process. The treated levels were T11 (n=6), T12 (n=49), L1 (n=48), L2 (n=21), L3 (n=7), L4 (n=28) and L5 (n=1). These patients were divided into two groups according to whether or not two fluoroscopes (PHILIPS /BV Libra and PHILIPS /BV 25 Gold) were available simultaneously. While one of the two fluoroscopes was being used for other surgeries, only single fluoroscope could be available for PVP. Group-I was composed of 60 patients who underwent PVP under the guidance of one C-arm fluoroscope. Group-II was consisted of 83 patients who underwent PVP under the guidance of two C-arm fluoroscopes. 

**Fig.1:**
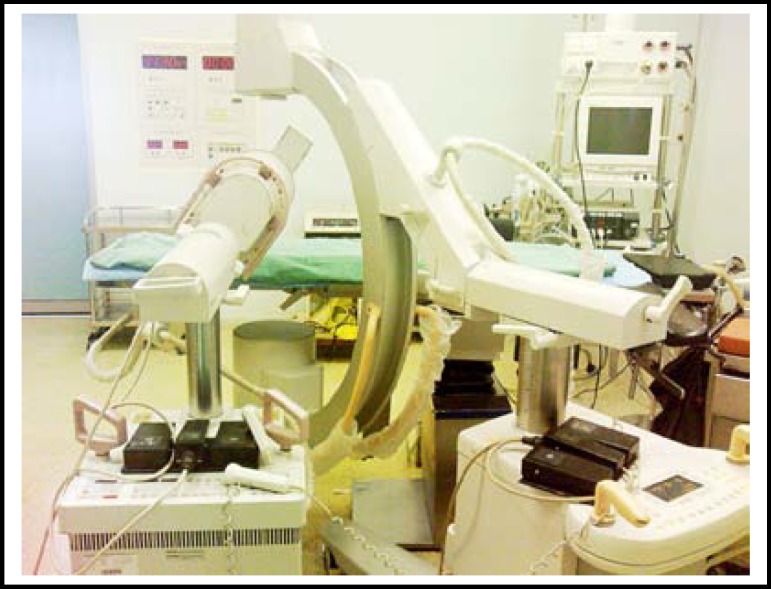
Placement of two C-arm fluoroscopes(lateral view)

**Fig.2:**
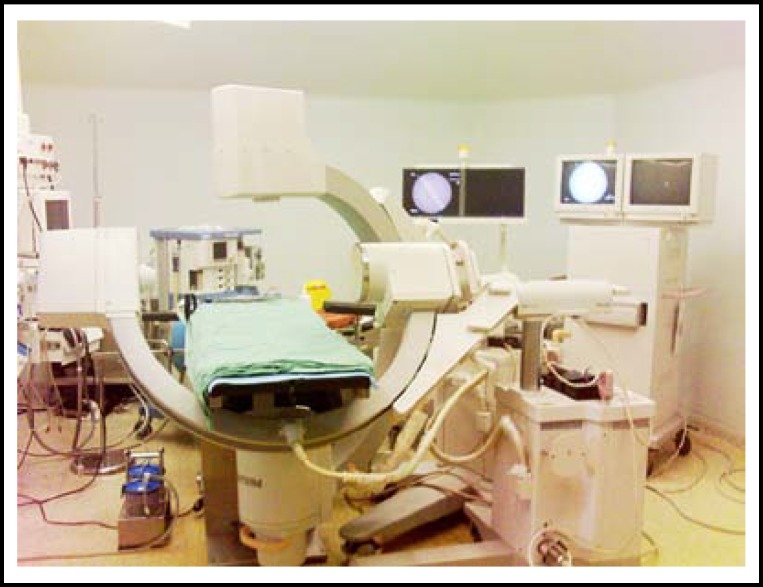
Placement of two C-arm fluoroscopes(front view

**Fig.3:**
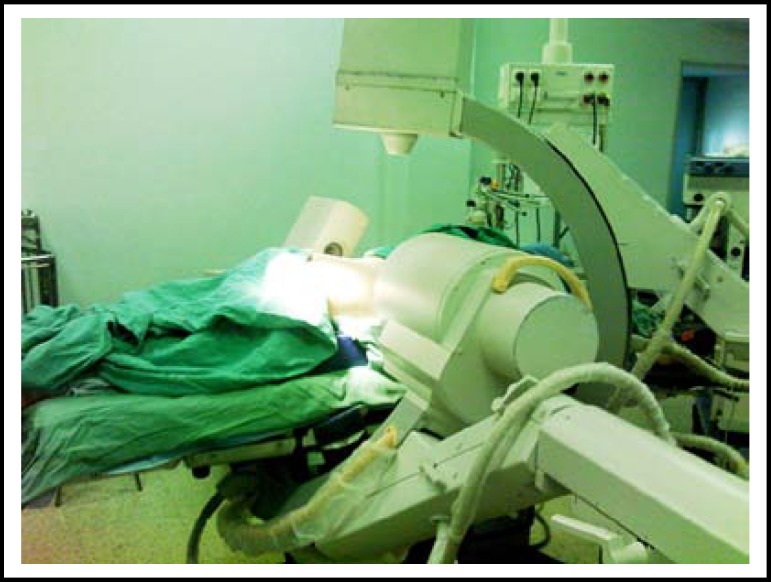
The patient was placed far away from the table column, allowing the positioning of the two fluoroscopes

The operations were performed in patients without contraindication. For Group 2, the two C-arm fluoroscopes were placed on the same side. The AP fluoroscope was positioned vertically with its plane tilted 45° to the long axis of the operation table. The lateral fluoroscope was placed horizontally with the C-arm under the operation table and its plane tilted close to the table, thus preventing collision with the C-arm of the AP fluoroscope (Fig. 1 and 2). PVP was performed under local anesthesia. The patient was placed far away from the table column (Fig.3), allowing the positioning of the two fluoroscopes. A single dose of preoperative antibiotic medication was administered 30 minutes before the start of the procedure to prevent infection. In order to reduce the exposure time, pulsed imaging was used to guide the procedure. After small incision of the skin an 11-gauge needle was placed percutaneously into the vertebral body. A transpedicular route was used in the lumbar spine, and an extrapedicular route in the thoracic spine. A large-bore needle was placed into the vertebral body under radiological guidance and acrylic cement (most commonly polymethylmethacrylate/PMMA) was then injected into the vertebral body until cement reaches the cortical wall of the vertebral body.

The operation time for each procedure was recorded from the local anesthesia to the removal of the trocar needle. The incidence of cement leakage was evaluated by reviewing the postoperative radiographs. The number of pulsed imagings, the time of operation and the incidence of cement leakage for both groups were recorded.

Statistical analyses were carried out using the SPSS statistical package, version 13.0(SPSS Inc., Chicago, IL, USA). Data were expressed as mean±SD. The number of pulsed imagings, the time of operation were compared between groups with Student’s t-tests. If there is non-normality or heteroskedasticity, Wilcoxon rank-sum test can be used. The incidence of cement leakage was compared between groups with chi-square test. A probability value of less than 0.05 was considered significant.

## RESULTS

The operations were all successfully accomplished. Pain was sufficiently relieved after PVP. The average number of pulsed imagings was 16.00±1.58 vs 13.07±2.00 per patient under the guidance of one vs two C-arm fluoroscopes. The average time of operation was 48.42±5.00 minutes vs 39.70±7.42 minutes per patient under the guidance of one vs two C-arm fluoroscopes. The incidence of cement leakage was 20% vs 15.7% of the patients under the guidance of one vs two C-arm fluoroscopes. The differences in the number of pulsed imagings and the time of operation were statistically significant. The difference in incidence of cement leakage was not statistically significant.

**Table-I:** Patient demographic data of both groups

	*Group 1 (one C-arm fluoroscope) (n=60)*	*Group 2 (two C-arm fluoroscopes) (n=83)*	*T*	*p value*
The number of pulsed imagings	16.00±1.58	13.07±2.00	9.40	0
The time of operation(minutes)	48.42±5.00	39.70±7.42	7.89	0
The incidence of cement leakage	20%	15.7%	^2^=0.454	0.5

## DISCUSSION

The vertebrae is surrounded by many important neurovascular structures. PVP requires the use of high-quality, high-resolution fluoroscopy for anteroposterior (AP) and lateral views. During the procedure guided by a single C-arm X-ray machine, the C-arm X-ray machine need to be rotated for either antero-posterior (AP) or lateral radiographic view, that might be time-consuming and may cause problems in maintaining sterile conditions, possibly increasing the infection rate. Projection realignment means more radiation exposure. More seriously, to take different radiographic views, the C-arm might take no less than several seconds to rotate, possibly causing delay in detecting cement leakage. Biplane fluoroscopy is preferred because it allows orthogonal, real-time visualization for cannula introduction and cement injection. However, this equipment is expensive and not commonly available in hospitals in China. An alternative is to use two C-arm fluoroscopes.

In this study, we found that the mean operation time in Group 2 under the guidance of two C-arm fluoroscopes was shorter than that of Group 1. A reduction of exposure time could have been achieved through pulsed imaging.^4^ In this study, we found that the average number of pulsed imagings in Group 2 was less than that of Group 1. Once the two fluoroscopes have been optimally settled down, it is maintained throughout the procedure and no radiation is wasted during readjusting. The two-fluoroscopic technique reduce decrease radiation exposure. That is second advantage of using the two-fluoroscopic technique. After the positioning of the two fluoroscopes, the surgeon can control the exposure via foot switches and finish the operation all by himself. That is another advantage of using the two-fluoroscopic technique. Cement leakage into paravertebral tissues is commonly reported in the literature.^5^^-^^7^ Care is therefore required when injecting cement. The difference in the incidence of cement leakage between both groups was not significant. The two C-arm fluoroscopic technique enables orthogonal visualization of injection, enabling paravertebral cement leakage to be noticed in time.

Our study had several limitations. This is not a randomized control trial. This is a non-standardized single-center study with a relatively small number of patients. We did not measure the radiation exposure time and the radiation dose.

## Authors Contributions:

**Ren-Jie Xu** **:** Conception; Statistical Analysis: Design, Execution, Review and Critique; Writing the first draft.

**Yong-Qing Yan:** Co-first-authors; Conception; Statistical Analysis: Design, Execution, Review and Critique; proofread.

**Guang-Xiang Chen, Tian-Ming Zou, Xiao-Qiang Cai:** Execution; Statistical Analysis: Execution; Critical Review of Manuscript.

**Dong-Lai Wang:** Conception and Organization; Statistical Analysis: Review of Manuscript.

## Notes

***Disclaimer***: There was no external funding in the preparation of this manuscript.

## References

[1] Johnell O, Kanis JA (2006). An estimate of the worldwide prevalence and disability associated with osteoporotic fractures. Osteoporos Int.

[2] Kanis JA, McCloskey EV, Johansson H, Strom O, Borgstrom F, Oden A (2008). Case finding for the management of osteoporosis with FRAX--assessment and intervention thresholds for the UK. Osteoporos Int.

[3] Cooper C, Atkinson EJ, Jacobsen SJ, O'Fallon WM, Melton LR (1993). Population-based study of survival after osteoporotic fractures. Am J Epidemiol.

[4] Boszczyk BM, Bierschneider M, Panzer S, Panzer W, Harstall R, Schmid K (2006). Fluoroscopic radiation exposure of the kyphoplasty patient. Eur Spine J.

[5] Chen JK, Lee HM, Shih JT, Hung ST (2007). Combined extraforaminal and intradiscal cement leakage following percutaneous vertebroplasty. Spine (Phila Pa 1976).

[6] Heini PF, Walchli B, Berlemann U (2000). Percutaneous transpedicular vertebroplasty with PMMA: operative technique and early results. A prospective study for the treatment of osteoporotic compression fractures. Eur Spine J.

[7] Prymka M, Puhler T, Hirt S, Ulrich HW (2003). Extravertebral cement drainage with occlusion of the extradural venous plexus into the vena cava after vertebrobplasty. Case report and review of the literature. Unfallchirurg.

